# Life‐threatening airway obstruction due to retropharyngeal and cervicomediastinal hematomas following transjugular intrahepatic portosystemic shunt procedure for acute variceal bleeding in cirrhosis.

**DOI:** 10.1002/ccr3.7005

**Published:** 2023-03-07

**Authors:** Long Li, Yong Chen

**Affiliations:** ^1^ Division of Interventional Radiology, Department of Medical Imaging Guangzhou Twelfth People's Hospital Guangzhou Guangdong China; ^2^ Division of Vascular and Interventional Radiology, Department of General Surgery, Nanfang Hospital Southern Medical University Guangzhou Guangdong China

**Keywords:** airway obstruction, coagulopathy, percutaneous internal jugular vein catheterization, retropharyngeal hematoma, transjugular intrahepatic portosystemic shunt, vascular complications

## Abstract

We report a case of life‐threatening airway obstruction due to retropharyngeal‐cervicomediastinal hematomas following transjugular intrahepatic portosystemic shunt or acute variceal bleeding in cirrhosis. Even though this is a rare complication, clinicians should maintain a high index of suspicion and evaluate and treat it promptly to prevent a fatal outcome.

## Abstract 2

Clinicians should be alert to the possibility of retropharyngeal and cervicomediastinal hematomas following percutaneous internal jugular vein catheterization in patients with cirrhosis and hypersplenism.

## 1 INTRODUCTION

Percutaneous internal jugular vein catheterization has become a widely accepted method in clinical practice for monitoring central venous pressure, administering fluids or drugs, and implementing parenteral nutrition and hemodialysis.^1^ Furthermore, internal jugular vein is a common vascular access in the field of interventional radiology, such as for creation of transjugular intrahepatic portosystemic shunt (TIPS) for treating portal hypertension.^2^ Although formation of a hematoma due to the bleeding complication at this puncture site is rare,^3^, ^4^ many unusual case reports are found in the literature.^5^, ^6^, ^7^, ^8^ Such hematomas could occur in the cervical,^5^, ^6^, ^9^, ^15^, ^18^, ^20^ retropharyngeal,^10^, ^11^, ^12^, ^16^ or mediastinal^7^, ^13^, ^14^, ^17^, ^19^, ^21^, ^22^, ^23^ regions, with the presentation varying widely, from asymptomatic^9^, ^11^, ^17^, ^18^, ^20^, ^21^ to life‐threatening.^5^, ^6^, ^7^, ^12^, ^13^, ^15^, ^16^, ^19^, ^23^ However, it is extremely rare that the hematoma simultaneously involves the retropharyngeal and cervicomediastinal regions, and causes life‐threatening airway obstruction after internal jugular vein catheterization.^24^, ^25^ We report a rare and unique case that presented with life‐threatening airway obstruction due to retropharyngeal and cervicomediastinal hematomas following TIPS procedure for acute variceal bleeding in cirrhosis.

## 2 CASE PRESENTATION

A 69‐year‐old man was admitted to the emergency department with sudden onset of an episode of hematemesis of 1000 mL. The patient was diagnosed more than 8 years ago as the decompensated stage of chronic viral hepatitis B–induced liver cirrhosis, complicated with severe gastroesophageal varices, hypersplenism, and ascites. Ten days before, he underwent endoscopic variceal ligation and endoscopic variceal obliteration with tissue glue due to hematemesis and melena.

On admission, initial examination revealed body temperature of 36.4°C, respiratory rate of 28 breaths/min, pulse rate of 114 beats/min, and blood pressure of 105/58 mm Hg. His blood routine test revealed the white blood cell (WBC) count of 3.13 × 10^9^/L with 77.1% neutrophils, red blood cell (RBC) count of 4.51 × 10^12^/L with the hemoglobin (HGB) level of 112 g/L, and platelet (PLT) count of 66 × 10^9^/L. Coagulation function test showed prothrombin time (PT) of 15.2 s (reference range, 11.0–14.5 s), international normalized ratio (INR) of 1.34 (reference range, 0.82–1.15), activated partial thromboplastin time (aPTT) of 38.8 s (reference range, 23.0–45.0 s), aPTT ratio of 1.33 (reference range, 0.82–1.15), thrombin time (TT) of 19.5 s (reference range, 14.0–21.0 s), and fibrinogen (FIB) of 1.01 g/L (reference range, 2.0–4.0 g/L). The liver function and renal function tests were normal. Emergent gastroscopy showed active bleeding from gastric body varices, and he was immediately subjected to endoscopic variceal ligation.

**FIGURE 1:**
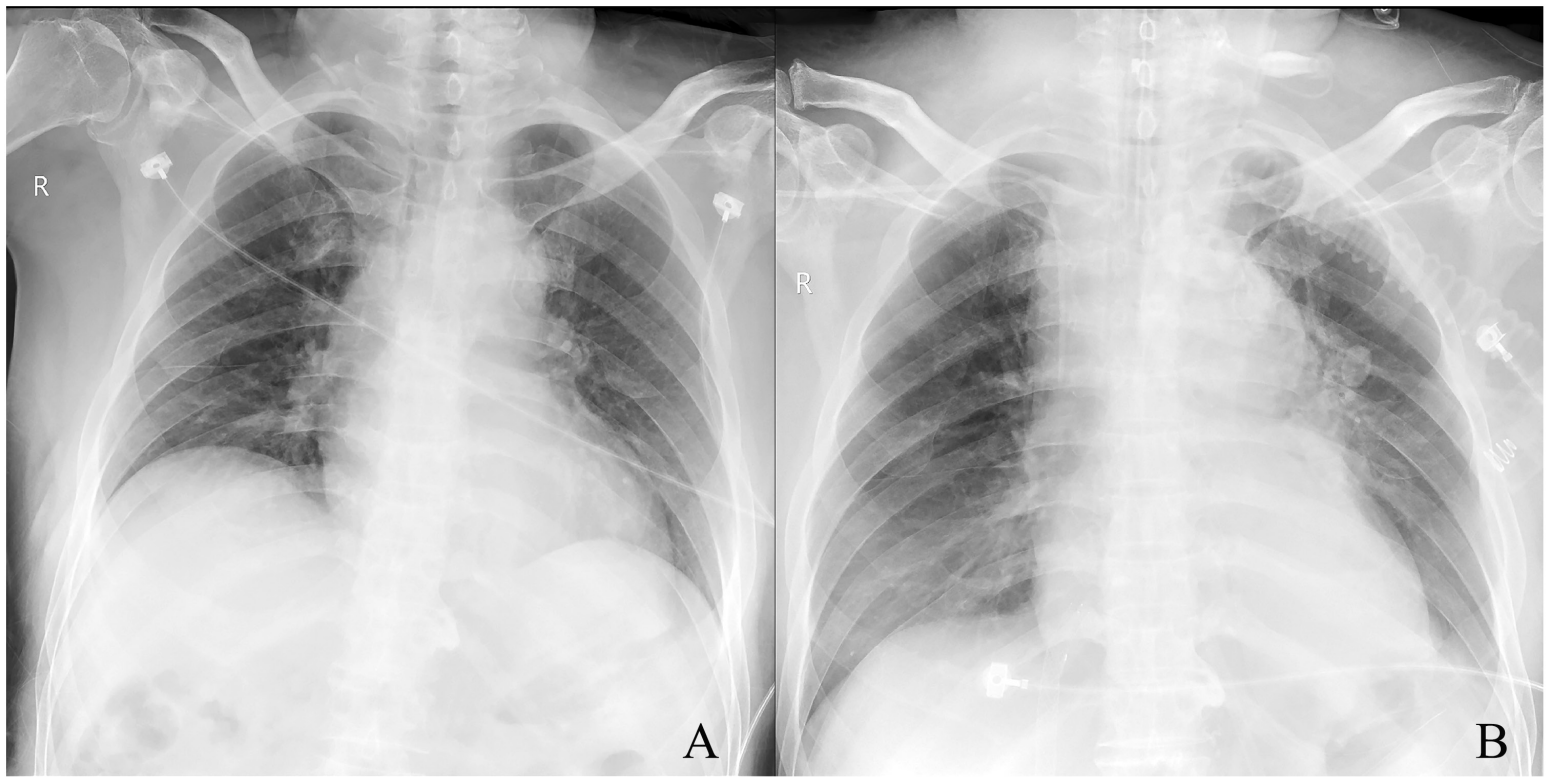
The bedside chest radiographs. (A), a bedside chest radiograph on admission showed no significant abnormalities. (B), the follow‐up bedside chest radiograph at 6 h after TIPS procedure demonstrated that the right neck root region and superior mediastinum widened.

On Day 1 after admission, the patient still defecated dark red‐colored bloody stool for three times, with the total amount of 550 mL. His blood pressure ranged from 91/67 mm Hg to 123/75 mm Hg, and the pulse rate fluctuated between 68 beats/min and 83 beats/min. His blood routine test showed the WBC count of 5.03 × 10^9^/L with 77.2% neutrophils, RBC count of 3.67 × 10^12^/L with HGB level of 86 g/L, and PLT count of 88 × 10^9^/L. Bedside chest radiography showed no significant abnormalities (Figure 1A). The patient was intended to be treated by TIPS, and was transferred to the Department of Interventional Radiology.

On Day 3 after admission, a TIPS procedure was performed via the right jugular vein under local anesthesia. Ultrasound‐guided right internal jugular vein puncture with Seldinger technique was performed with a Neff Percutaneous Access Set (NPAS‐100‐RH‐NT G10544) (Cook Medical, Bloomington), composed by a 22‐gauge needle, a 0.018‐inch (0.045‐cm) guidewire, a 4‐Fr coaxial dilator, and a 6‐Fr sheath. Specifically, the right internal jugular vein puncture using the Seldinger technique under ultrasound guidance in the first attempt was performed with a 22‐gauge needle of the Neff Percutaneous Access Set (NPAS‐100‐RH‐NT G10544) (Cook Medical, Bloomington, IN, USA). After free aspiration of blood, a 0.018‐inch (0.045‐cm) guidewire was threaded freely through the needle without resistance, and a 4‐Fr coaxial dilator and a 6‐Fr sheath were passed over the guidewire without any resistance. Under fluoroscopy guidance, a 0.035‐inch Terumo guide wire (Terumo Corp) was guided into the inferior vena cava via the 6‐Fr introducer. The introducer site was dilated with an 8‐Fr dilator. A Rösch‐Uchida Transjugular Liver Access Set (RUPS‐100 G06929) (Cook Medical, Bloomington) with a 10‐Fr introducer was used to puncture the portal vein via the distal hepatic vein. The systemic and portal vein pressures were measured through the portosystemic access, and the portosystemic pressure gradient was 25 cm H_2_O. The 0.035‐inch Terumo guide wire and a 5‐Fr Cordis Cobra catheter (Cordis Corp) were used to traverse the gastric varices. A 25% mixture of N‐butyl‐2‐cyanoacrylate (NBCA) (Beijing Compont Medical Devices Co., Ltd) and Lipiodol was injected through the microcatheter with its tip in the varices by use of a continuous single‐column injection technique until all of the varices were opacified with the mixture.

When a balloon dilatation catheter was introduced, the guidewire was withdrawn carelessly from the portosystemic access. Then, the portal vein had to be punctured again. The tract between the hepatic and portal veins was dilated using a 6 mm × 80 mm balloon dilatation catheter (Cook Medical). A portosystemic shunt was created using an 8 mm × 70 mm GORE® VIATORR® TIPS Endoprosthesis (W. L. Gore & Associates, Inc) and an 8 mm × 80 mm Vascular Self‐Expanding Stent (Cook Medical), and dilated using an 8 mm × 80 mm balloon dilatation catheter (Cook Medical). The systemic and portal vein pressures were measured through the portosystemic shunt, and the portosystemic pressure gradient was 25 cm H_2_O. The 10‐Fr introducer was withdrawn, and hemostasis of the right internal jugular vein puncture site was achieved by manual compression for 15 min. The total procedural time was about 2 h. When the patient returned to the hospital ward, his vital signs were as follows: respiratory rate, 13 breaths/min; pulse rate, 60 beats/min; blood pressure, 118/74 mm Hg; blood oxygen saturation, 100%.

At 6 h after TIPS procedure, the patient complained of chest tightness and shortness of breath. His right side of the neck appeared swollen. No thrill was palpated or auscultated around the puncture site. Vital signs showed blood pressure of 106/55 mm Hg, pulse rate of 62 beats/min, respiratory rate of 20 beats/min, and oxygen saturation of 100% on room air. The patient was conscious and responsive. Emergency bedside chest radiograph demonstrated that the right neck root region and superior mediastinum widened (Figure 1B). Emergency bedside ultrasound revealed subcutaneous soft tissue swelling around the right neck puncture site, no abnormal mass, and no markedly abnormal findings of the carotid artery and jugular vein.

His blood routine test revealed the WBC count of 9.72 × 10^9^/L with 88% neutrophils, RBC count of 3.80 × 10^12^/L with HGB level of 91 g/L, and PLT count of 80 × 10^9^/L; coagulation function test showed PT of 16.4 s, INR of 1.45, aPTT of 31.5 s, aPTT ratio of 1.44, TT of 24.6 s, and FIB of 0.45 g/L. The related equipment for emergency trachea intubation was readied.

**FIGURE 2:**
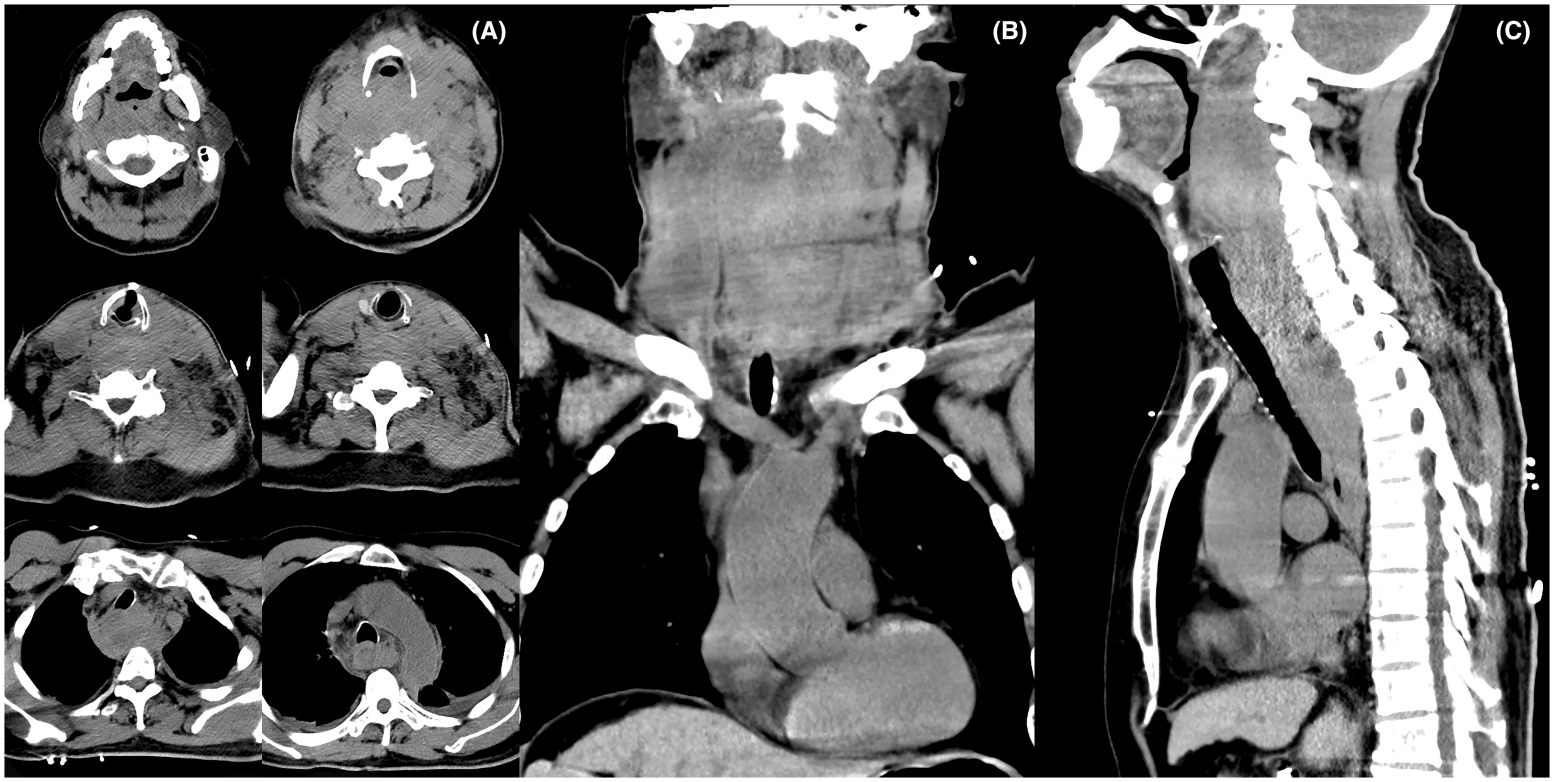
CT images at 4 h after the onset of dyspnea. CT plain scan showed neck swelling, blurred fat space, subcutaneous edema in anterior cervical region, a soft tissue density mass along the back of the esophagus from the level of the mandibular angle to posterior mediastinum below the level of tracheal bifurcation, and narrowing of oropharynx and laryngopharynx. (A), axial images; (B), coronal reformation image; (C), sagittal reformation image.

At 10 h after TIPS procedure, plain computed tomography (CT) scan showed hematoma extending from the nasopharyngeal region and right side of the neck all the way down to the posterior mediastinum at the level of the aortic arch (Figure 2). When contrast‐enhanced CT scan was about to begin, the patient suddenly experienced dysphoria, lapsed into unconsciousness, and developed cyanosis of his lips and distal extremities. His breathing and heart stopped. External chest compression, tracheal intubation, and ventilator‐assisted breathing were performed immediately. After 5 min, he experienced ventricular fibrillation. After nonsynchronized cardioversion was performed three times with an energy setting of 200 J, his heart rate and spontaneous breathing recovered (blood pressure, 146/62 mm Hg; pulse rate, 100 beats/min; and oxygen saturation, 100% on room air), but remained in a deep coma.

The patient was transferred to the Intensive Care Unit for further management. At 11 hours after TIPS procedure, he was subjected to intravenous sedation, mild hypothermic neuroprotection, decrease of cerebral edema with mannitol, and antiepileptic therapy with sodium valproate.

At 11 h after TIPS procedure, his blood routine test showed the WBC count of 12.28 × 10^9^/L with 94.9% neutrophils, RBC count of 2.91 × 10^12^/L with HGB level of 72 g/L, and PLT count of 67 × 10^9^/L; coagulation function test showed PT of 20.9 s, INR of 1.86, aPTT of 52.0 s, aPTT ratio of 1.83, TT of 27.4 s, and FIB of 0.27 g/L.

At 17 h after TIPS procedure, his blood routine test showed the WBC count of 14.00 × 10^9^/L with 93.8% neutrophils, RBC count of 2.58 × 10^12^/L with HGB level of 66 g/L, and PLT count of 51 × 10^9^/L; coagulation function test showed PT of 23.2 s, INR of 2.06, aPTT of 56.0 s, aPTT ratio of 2.04, TT of 33.6 s, and FIB of 0.23 g/L. He was given intravenous blood transfusion.

At 30 h after TIPS procedure, which was 24 h after the onset of dyspnea, follow‐up CT showed no significant change in the extent of the cervicomediastinal hematoma. Emergency surgical repair was suggested but declined by the patient family.

After the patient successively received 11 units of packed RBCs, 1500 mL of fresh frozen plasma, and 2 units of PLT, his hemogram and coagulation function improved progressively. At 54 h after TIPS procedure, his blood routine test showed the WBC count of 8.89 × 109/L with 94.4% neutrophils, RBC count of 2.99 × 1012/L with HGB level of 80 g/L, and PLT count of 48 × 109/L; coagulation function test showed PT of 15.4 s, INR of 1.36, aPTT of 40.9 s, aPTT ratio of 1.35, TT of 17.1 s, and FIB of 1.81 g/L. The patient remained in a persistent coma state.

At 8 days after admission, which was 5 days after TIPS procedure, his family refused further treatment, and the patient was discharged due to the poor prognosis of the disease.

## 3 DISCUSSION

This elderly male patient experienced acute asphyxia and persistent deep coma that resulted from retropharyngeal and cervicomediastinal hematomas, which spread from the internal jugular vein puncture site after TIPS procedure for acute variceal bleeding in the setting of cirrhosis and hypersplenism. Dyspnea developed 6 h after TIPS procedure; acute asphyxia occurred 10 h after TIPS procedure; and then, the patient presented with persistent deep coma. The HGB level decreased from 112 g/L before TIPS procedure to 66 g/L at 17 h after TIPS procedure, and stabilized around 80 g/L until 11 units of packed RBCs were infused intravenously at 54 h after TIPS procedure. Although the coagulation test results were generally normal on admission, coagulation hypofunction occurred after hematoma formation and did not return to the normal level until 1500 mL of fresh frozen plasma was infused intravenously at 54 h after TIPS procedure. Low blood PLT count was found throughout the course of the disease. In this patient, puncture was made under ultrasonographic guidance, which confirmed that the 22‐gauge puncture needle entered the right internal jugular vein. It is not possible to determine which vessel was responsible for the hematoma, but its size and the speed at which it enlarged suggest that the hematoma resulted from the puncture site of the right internal jugular vein.

Vascular complications of internal jugular vein catheterization by insertion site may be due to several reasons such as inexperience, the number of attempts at needle insertion, the use of relatively larger gauge needle, severe dehydration, morbid obesity, and coagulopathy.^23^ Inadvertent arterial injury during internal jugular vein catheterization is the most common cause of hematoma formation at this puncture site. The most frequent site of arterial injury is the carotid artery. Other reported sites of injury include the innominate (brachiocephalic) artery, subclavian artery, aortic arch, descending thoracic aorta, vertebral artery, thyroid artery,^6^, ^25^, ^26^, ^27^, ^28^, ^29^ transverse cervical artery,^30^ and thyrocervical trunk.^31^, ^32^ The arterial injury occurs when the puncture needle goes through the internal jugular vein into the carotid artery, or the needle penetrates the artery that is very close to the internal jugular vein, or the guidewire travels through the internal jugular vein and its posterior wall into the carotid artery.^6^, ^26^, ^33^ The use of ultrasound guidance during internal jugular vein catheterization has been proposed as a standard technique to reduce the risk of arterial cannulation and increase the rate of successful vein insertion.^34^, ^35^ To increase the success rate of ultrasound‐guided vascular access procedures, the short‐axis view with the out‐of‐plane technique should be used to advance the needle tip to the internal jugular vein; the long‐axis view with the in‐plane technique should be used to rupture the anterior wall; and the short‐axis view and the coronal view from the supraclavicular fossa should be used to confirm the guidewire's position from the internal jugular vein to the brachiocephalic vein.^34^, ^35^, ^36^ The Trendelenburg position or 45‐degree passive leg raise, Valsalva maneuver, or liver compression are used to increase the diameter and the dimension of the internal jugular vein, and can help ultrasound to better visualize the internal jugular vein.^36^, ^37^ The traditional method for detecting arterial injury is to observe the color and pulsatile or high‐pressure backflow coming from the needle or sheath. However, this method has been shown to be unreliable.^6^, ^26^, ^27^, ^28^, ^29^, ^33^, ^34^, ^35^, ^36^, ^37^ During the TIPS procedure, the method for identifying arterial injury is angiography obtained with the injection of low doses of contrast medium via the needle or sheath. If a needle thinner than 14 G (equivalent to a diameter of 2.1 mm) or a catheter (or a dilator) smaller than 7 Fr (equivalent to a diameter of 2.3 mm) is inserted into an artery, pulling the needle or the dilator from the artery and applying external pressure (the “pull‐and‐pressure” approach) to prevent hemorrhagic complications is a common management approach, which seems inconsequential in most cases.^6^, ^26^, ^27^, ^28^, ^29^, ^33^, ^34^, ^35^, ^36^, ^37^ It is worth noting that the prolonged carotid artery compression could cause ischemic stroke.^6^, ^26^, ^27^, ^28^, ^29^, ^33^, ^34^, ^35^, ^36^, ^37^ However, if a catheter (or a dilator) larger than 7 Fr is inserted into an artery, particularly in the carotid artery, the pull‐and‐pressure approach is associated with a significant risk of stroke, hemothorax, pseudoaneurysm formation, airway‐compromising hematoma, and arteriovenous fistula.^29^ The suggested algorithm for the management of inadvertent arterial injury during internal jugular vein catheterization includes leaving the dilator or catheter in situ in the artery while management preparations are made, and endovascular treatment with balloon tamponade, percutaneous closure devices, covered stent grants, and embolization offer good results when selected appropriately based on imaging evaluation.^6^, ^26^, ^28^, ^29^, ^36^ After arterial repair, prompt neurological evaluation should be performed, even if it requires postponing elective intervention.^6^, ^26^, ^28^, ^29^, ^36^

Inadvertent venous injury during internal jugular vein catheterization has been reported to occur in the puncture site, subclavian vein, superior vena cava, innominate–superior vena cava junction, azygos vein, and right atrium.^7^, ^14^, ^19^, ^23^, ^33^ The venous injury may occur when the guidewire becomes stuck in the vessel wall and subsequent insertion of a dilator or a catheter causes a linear laceration, or the dilator or catheter is advanced violently without the guidewire guidance.^23^, ^26^, ^33^ Fluoroscopy guidance can be used to identify the anatomic location of the guidewire and observe the entire course of the guidewire as it is advanced so as to ensure that it follows a proper anatomic pathway and is not trapped against the vein.^26^, ^33^ Fluoroscopy can also be used to observe the real‐time course of dilators and catheters as they are advanced into the venous system, and help to prevent injuries to veins.^26^ During the TIPS procedure, it should be emphasized that the introducer can be advanced via the guidewire until the guidewire has been detected in the inferior vena cava.

Coagulopathy is a considerable risk factor for hematoma formation in the vascular access site.^37^ The GAVeCeLT consensus statement published by the Italian multidisciplinary group for venous access devices in 2022 recommends the following: For patients with disease‐induced coagulopathy with PT/INR >1.5 and/or aPTT ratio >1.3, there is no contraindication to minimally invasive procedures (all peripheral venous access devices, nontunneled peripherally inserted central catheters, nontunneled femorally inserted central catheters at mid‐thigh); there is relative contraindication to moderately invasive procedures (nontunneled central inserted central catheters, nontunneled femorally inserted central catheters at the groin, tunneled peripherally inserted central catheters, nontunneled dialysis catheters); and there is absolute contraindication to highly invasive procedures (tunneled central inserted central catheters, tunneled femorally inserted central catheters, tunneled‐cuffed dialysis catheters, ports and peripherally inserted central catheter‐ports). For patients with platelet count <50 × 10^9^/L, there is no contraindication to minimally invasive procedures; there is relative contraindication to moderately invasive procedures; and there is absolute contraindication to highly invasive procedures.^38^ According to the characteristics of transjugular vein catheterization (the size and location of the vein, the diameter of the introducer used, the difficulty of venous cannulation, and the feasibility of compressing maneuvers for reducing the bleeding), TIPS should be considered a highly‐invasive venous‐access procedure mentioned by the GAVeCeLT consensus statement. This patient had a definite history of hypersplenism caused by liver cirrhosis, aPTT ratio of 1.33 before the TIPS procedure, and the persistent coagulation disorder after the TIPS procedure. Therefore, the coagulation disorder caused by liver cirrhosis should be considered the primary cause that resulted in the retropharyngeal and cervicomediastinal hematomas in this patient.

Taken together, hematoma formation at the puncture site in this patient should be considered in relation to the known coagulation disorder based on cirrhosis and the TIPS procedure taking longer than 2 h. For those situations, it is reasonable to remove the 10‐Fr sheath with a delay until the coagulation disorder has been confirmed to improve, or a percutaneous vascular closure device should be used when the 10‐Fr sheath is removed.

The retropharyngeal space is an anatomical region between visceral fascia and alar fascia of the deep layer of the deep cervical fascia, and spans from the base of the skull to the mediastinum. Its location is anterior to the prevertebral muscles, and posterior to the nasopharynx, oropharynx, hypopharynx, larynx, trachea, and esophagus. It is bounded anteriorly by the buccopharyngeal fascia, laterally by the carotid sheath, posteriorly by the prevertebral fascia, and inferiorly by the alar fascia, which fuses with the middle layer of the deep cervical fascia, typically around the T4 vertebral body.^39^ The “danger space” of the retropharyngeal space is located between the alar and prevertebral divisions of the deep layer of the deep cervical fascia, immediately posterior to the retropharyngeal space and immediately anterior to the prevertebral space, and extends from the skull base to the posterior mediastinum and diaphragm.^39^ Effusion including hemorrhage or infection in the retropharyngeal space may push forward and occlude the airway at the level of the pharynx, appearing as anterior displacement of one or both sides of the posterior pharyngeal wall due to the midline fascial raphe. Furthermore, effusion can spread inferiorly via the danger space to the mediastinum, and the spread within the danger space tends to occur rapidly because of the loose areolar tissue that occupies this region.^39^ In this patient, the internal jugular vein bleeding entered into the carotid sheath, and then spread within the retropharyngeal space and mediastinum.

Retropharyngeal hematoma can obstruct the airway to cause lethal consequences. Respiratory symptoms usually indicate airway obstruction. Common symptoms in patients with retropharyngeal hematoma are dyspnea, dysphagia, neck pain, stridor, hoarseness, altered mental state, neck swelling, and cyanosis.^40^ A lateral soft tissue radiograph of the neck shows widening of the retropharyngeal area, the prevertebral area, or both. Contrast‐enhanced CT scanning of the neck and chest should be considered the investigation of choice because it is helpful in showing the level of obstruction and the extent of the hematoma, identifying the source of bleeding, and making the planning of anesthesia and surgery. A tracheotomy under local anesthesia may be the only safe option, as intubation with a small‐lumen tube may not pass the inferior limit of the hematoma. A rough oropharyngeal intubation attempt can end in hematoma rupture, with bleeding and escalating edema, further compromising the airway. When the hematoma continues to expand or when ventilation is difficult despite the tracheotomy, surgical evacuation of the hematoma is indicated.^40^ Surgery and transarterial embolization are commonly used to control active bleeding in patients with retropharyngeal hematoma. The standard‐of‐care for this complication is neck exploration with surgical repair.^41^, ^42^ Advantages of transarterial embolization are as follows: not necessarily needing general anesthesia, rapid access, short procedure time, ability to control multiple local bleeding points, and ability to use angiography to localize superior hemorrhage origin and perform super‐selective therapeutic vessel occlusion by cannulating the smaller vessel branches not amenable to open surgical repair.^6^, ^26^, ^28^, ^29^, ^36^

## 4 CONCLUSIONS

To the best of our knowledge, this is the first case report describing life‐threatening airway obstruction due to retropharyngeal and cervicomediastinal hematomas following TIPS procedure for acute variceal bleeding in cirrhosis. Though the case presented in this report is rare, clinicians should be alert to the possibility of retropharyngeal and cervicomediastinal hematomas following percutaneous internal jugular vein catheterization in patients with cirrhosis and hypersplenism.

## AUTHOR CONTRIBUTIONS

Yong Chen contributed to the design and implementation of the study. Long Li contributed to reviewing the literature and designing and writing the manuscript. Yong Chen established the diagnosis and revised the manuscript critically for important intellectual content. All authors read and approved the final version of this manuscript for submission.

## FUNDING INFORMATION

There is no funding to present the essay.

## CONFLICT OF INTEREST

No conflict of interest is declared by the authors.

## ETHICAL APPROVAL

The authors all declare that this manuscript is not published or under consideration in other journals.

## CONSENT

Written informed consent has been acquired from the daughter of the patient to publish this case report according to the journal's patient consent policy. Moreover, the authors all declare that patients' confidentiality has been respected.

## Data Availability

All data generated or analysed during this study are included in this published article.
